# The Churches and the War: Hospital Sunday

**Published:** 1918-06-22

**Authors:** 


### The Hospital

The Workers' Newspaper of Administrative Medicine and Institutional Life, Administration, National Insurance and Health.

No. 1672, Vol. LXIV. SATURDAY, JUNE 22, 1918. PRICE ONE PENNY

### THE CHURCHES AND THE WAR.

### Hospital Sunday.

The late Canon Miller's platform to unite the Churches of all denominations and sects on one day, as an act of thanksgiving on their own part and that of their congregations to Almighty God for the blessings of health especially, and other blessings too, throughout each year, should prove a living force indeed on Hospital Sunday, June 23, 1918. The war, by its awfulness, voluntary sacrifice, and rendering up of individual life in defence of their country, has awakened millions of our fighting men in all Services to the reality of their spiritual nature, to the presence and Fatherhood of Almighty God, and the acceptance of themselves by Him through Christ, whose battle they are fighting, in fact, in this war, with a certainty and conviction never before realised by humanity in the history of the world. No one who has had the privilege to risk his own life, whether warrior or civilian, in the course of the present war, can have failed, when at the Front, to realise the teaching, uplifting force and vitalising influence of the atmosphere of self-surrender, devotion, and inspiriting dependence on Almighty God, to be shared practically everywhere by those who have eyes to see and hearts alive to the realities of life and death. Hospital Sunday, if all who are defending the Homeland and its inhabitants could be brought back to Blighty on the 23rd instant, would cause the Places of Worship of all denominations to be crowded to the roof without a doubt.

If we had our way, we would arrange and secure that every clergyman and minister of religion throughout the British Isles should be sent out to the Front and passed through the camps, hospitals, ambulance stations, and base hospitals. They should also be taken into the firing-line, should be present at the burial of the dead warriors and murdered doctors, nurses, orderlies, and others; for then, indeed, every clergyman, minister, priest, and officer of religion would at length, in God's mercy, awaken to the realities of the life and faith of our warriors and of all men and women upon whose zealous and patriotic service their protection, and possibly their very existence, depends. At the moment it is grievous to know and sad to feel it is one's duty to record that, although there are many good men and true who are alive to their spiritual nature, who have shed conventionalities and made the services for which they are responsible in the House of God a real, living force in the Homeland, to-day, there are, alas, so many more men who exhibit no sense of being alive to their own spiritual nature or the spiritual natures and requirements of the people, for whose living faith and ultimate salvation they may one day be held responsible to Almighty God.

What we have here written is but the expression of the thoughts of an ever-increasing number of earnest-minded people in the Homeland whose misfortune it may be to be attached to a congregation, where the clergyman, minister or religious leader has throughout the war pursued conventional methods, and, still persisting in them, has forced the more earnest-minded amongst his congregation to go out into the fields or the parks, or to other Houses of God than their own, that they may have the cheer and uplifting comfort of feeling in their worship the stirring of their spiritual nature and their nearer approach to their Father and Saviour. This state of affairs cannot continue without disaster to the Churches, and, when our warriors return home, may involve the compulsory retirement of every minister of the conventional type and the substitution of a man of God, fired with the sense of his spiritual nature and his responsibilities for the quickening and culture of every member of his flock. For indeed our warriors are likely to force such a solution in their state of awakenment to their spiritual natures, realising that otherwise a wave of atheism may sweep the nation from end to end to its ruin and destruction.

Canon Miller, earnest and devout man as he was, full of zeal for the unity of the Churches on Hospital Sunday, we may believe will be with every minister and congregation throughout the Metropolis of the Empire on Hospital Sunday, the 23rd instant, and indeed with every community this year which numbers Hospital Sunday amongst its established privileges. Hospital Sunday in London this week may in God's mercy supply the awakening and vitalising force not only for congregations and people, but for every minister and religious teacher within its influence. It has already by the preliminary work, amongst many church communities and parishes, in preparation for Hospital Sunday, knit together the leaders of religious thought in combined efforts by house-to-house visitation, by organising collections throughout the streets and squares on Hospital Sunday, and no doubt by the preparation of special sermons, caused every such church and community to prepare for the return of our warriors, and the stirring to the depths of the spiritual nature of men and women, to an extent seldom equalled, and certainly never surpassed, since the war began.

The war has brought us the maroon and the bugle to warn, protect, arouse, and comfort the citizens throughout London. Hospital Sunday in the parishes and districts we have referred to has already sounded, or is prepared to sound, the bugle of awakenment to the spiritual nature of men and women in this vast city. In how many pulpits in the Metropolis of the Empire will the trumpet for which the ministers are responsible give an uncertain or no sound at all? The newspapers have made themselves very welcome to thoughtful people of late by devoting as much as a whole column each week to the names of churches, preachers and subjects each Sunday. We venture to hope our lay contemporaries this year will make it their business to inquire and record in next Saturday's issue, the 22nd instant, and in the Sunday papers in their issue of the 23rd instant, the names of the clergy of all denominations, with that of their churches, who are alive to their spiritual nature and that of their congregations. There the trumpet will sound in full blast to the renewal of hope and the comfort of all types and classes of Londoners possessing minds and intelligence.

